# Inhibition of a Putative Dihydropyrimidinase from *Pseudomonas aeruginosa* PAO1 by Flavonoids and Substrates of Cyclic Amidohydrolases

**DOI:** 10.1371/journal.pone.0127634

**Published:** 2015-05-19

**Authors:** Cheng-Yang Huang

**Affiliations:** 1 School of Biomedical Sciences, Chung Shan Medical University, No.110, Sec.1, Chien-Kuo N. Rd., Taichung City, Taiwan; 2 Department of Medical Research, Chung Shan Medical University Hospital, No.110, Sec.1, Chien-Kuo N. Rd., Taichung City, Taiwan; Institute of Enzymology of the Hungarian Academy of Science, HUNGARY

## Abstract

Dihydropyrimidinase is a member of the cyclic amidohydrolase family, which also includes allantoinase, dihydroorotase, hydantoinase, and imidase. These metalloenzymes possess very similar active sites and may use a similar mechanism for catalysis. However, whether the substrates and inhibitors of other cyclic amidohydrolases can inhibit dihydropyrimidinase remains unclear. This study investigated the inhibition of dihydropyrimidinase by flavonoids and substrates of other cyclic amidohydrolases. Allantoin, dihydroorotate, 5-hydantoin acetic acid, acetohydroxamate, orotic acid, and 3-amino-1,2,4-triazole could slightly inhibit dihydropyrimidinase, and the IC_50_ values of these compounds were within the millimolar range. The inhibition of dihydropyrimidinase by flavonoids, such as myricetin, quercetin, kaempferol, galangin, dihydromyricetin, and myricitrin, was also investigated. Some of these compounds are known as inhibitors of allantoinase and dihydroorotase. Although the inhibitory effects of these flavonoids on dihydropyrimidinase were substrate-dependent, dihydromyricetin significantly inhibited dihydropyrimidinase with IC_50_ values of 48 and 40 μM for the substrates dihydrouracil and 5-propyl-hydantoin, respectively. The results from the Lineweaver−Burk plot indicated that dihydromyricetin was a competitive inhibitor. Results from fluorescence quenching analysis indicated that dihydromyricetin could form a stable complex with dihydropyrimidinase with the *K*_d_ value of 22.6 μM. A structural study using PatchDock showed that dihydromyricetin was docked in the active site pocket of dihydropyrimidinase, which was consistent with the findings from kinetic and fluorescence studies. This study was the first to demonstrate that naturally occurring product dihydromyricetin inhibited dihydropyrimidinase, even more than the substrate analogs (>3 orders of magnitude). These flavonols, particularly myricetin, may serve as drug leads and dirty drugs (for multiple targets) for designing compounds that target several cyclic amidohydrolases.

## Introduction

Dihydropyrimidinase catalyzes the reversible cyclization of dihydrouracil or dihydrothymine to *N*-carbamoyl-β-alanine or *N*-carbamyl-β-aminoisobutyrate in the second step of the pyrimidine degradation pathway, respectively [1,2]. Dihydropyrimidinase, a component in the chain of pyrimidine catabolism, is also capable of detoxifying xenobiotics with an imide functional group that ranges from linear imides to heterocyclic imides [3,4,5] and organic cyclic carbonates [6]. These different imide-hydrolyzing enzymes from microorganisms are normally known as hydantoinase because of their role as biocatalysts in the synthesis of D- and L-amino acids for the industrial production of the precursors of antibiotics [7,8]. Although dihydropyrimidinase and hydantoinase generally have a similar active site, their overall sequence identity and substrate specificity differ [9]. Thus, some bacterial hydantoinases are still named and identified as dihydropyrimidinase because of the catalytic activity toward the natural substrates dihydrouracil and dihydrothymine.

**Fig 1:**
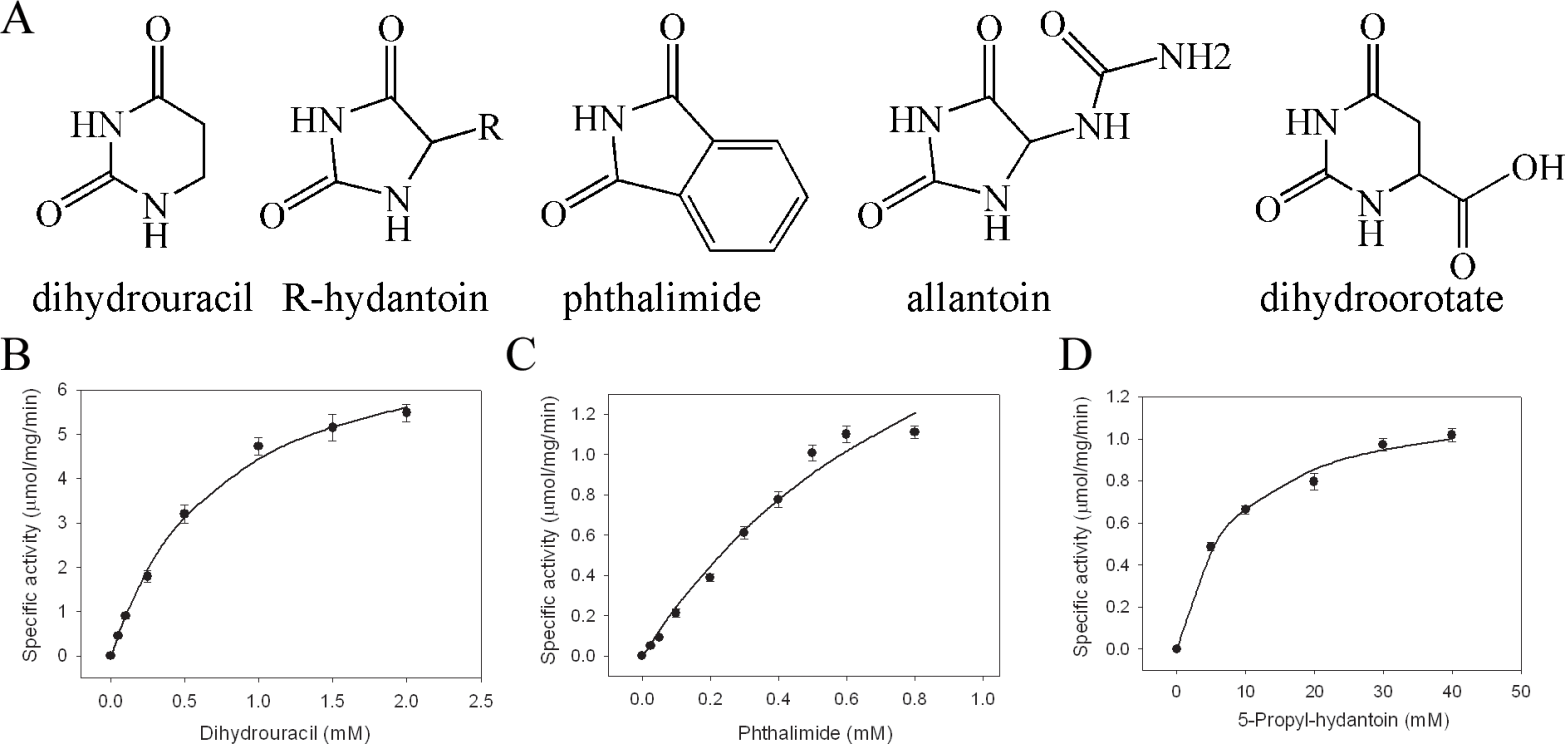
Substrate specificity of dihydropyrimidinase. (A) Substrate of dihydropyrimidinase, hydantoinase, imidase, allantoinase, and dihydroorotase. Kinetic analysis of dihydropyrimidinase was carried out using (B) dihydrouracil, (C) phthalimide, and (D) 5-propyl-hydantoin as a substrate. The maximal concentration of phthalimide was limited to 1 mM due to its poor solubility. Data points are an average of 2–3 determinations within 10% error.

Based on their functional and structural similarities, dihydropyrimidinase, hydantoinase, imidase, allantoinase, and dihydroorotase belong to the cyclic amidohydrolase family [10]. Even if these enzymes have similar functions, they have relatively low amino acid sequence identity. These metal-dependent enzymes catalyze the ring-opening hydrolysis of the cyclic amide bond of each substrate in either five- or six-membered rings in the metabolism of purines, pyrimidines, and many xenobiotics (Fig 1A) [9,11,12,13]. Almost all the active sites of dihydropyrimidinase, hydantoinase, allantoinase, and dihydroorotase contain four histidines, one aspartate, and one post-carboxylated lysine residue, which are required for metal binding and catalytic activity. The presence of a post-carboxylated lysine in hydantoinase is also required in binuclear metal center self-assembly [12], and it increases the nucleophilicity of the hydroxide for catalysis [14]. Previous studies indicated that cyclic amidohydrolases should use a nearly identical mechanism for catalysis. However, the substrate selectivity and specificity of dihydropyrimidinase, hydantoinase, allantoinase, and dihydroorotase highly differ. For example, dihydroorotase does not hydrolyze dihydropyrimidine, hydantoin, and allantoin [15]. Thus, whether the substrate and inhibitor of each enzyme in this family competitively inhibit dihydropyrimidinase remains unclear.

Few therapies are effective against the six antibiotic-resistant ESKAPE pathogens. The increased prevalence of beta-lactamases in *P*. *aeruginosa* and other bacteria has begun to reduce the clinical efficacy of beta-lactams against the most common opportunistic pathogen [16]. To date, over 800 beta-lactamases have been identified, of which at least 120 beta-lactamases have been detected in *P*. *aeruginosa* [17]. The development of clinically useful small-molecule antibiotics and identification of new targets in microorganisms are seminal events in the field of infectious diseases [18].

Flavonols belong to flavonoids, the most common group of plant polyphenols that is responsible for most of the flavor and color of fruits and vegetables [19]. Over 5,000 different flavonoids have been identified, many of which display structure-dependent biological and pharmacological activities [20,21,22], including antimicrobial agents [23,24]. These natural products are safe as pharmaceuticals because they have fewer side effects for human use.

In this study, we investigated the effects of the substrates and inhibitors of allantoinase and dihydroorotase, including the flavonols myricetin, quercetin, kaempferol, and galangin, on inhibiting the catalytic activity of a putative dihydropyrimidinase from *P*. *aeruginosa* PAO1. The derivatives of myricetin, namely, dihydromyricetin and myricitrin, were further used to test the structure–inhibition relationship of dihydropyrimidinase.

## Materials and Methods

### Construction of the dihydropyrimidinase expression plasmid

*PA0441*, the gene encoding the putative dihydropyrimidinase, was amplified by PCR using genomic DNA of *P*. *aeruginosa* genomic DNA as the template. The forward (5′-CGCGGCATATGTTTGATTTACTCCTGC-3′) and the reverse (5′-TCGCACTCGAGAAAATCGAAGGCATGT-3′) primers were designed to introduce unique NdeI and XhoI restriction sites (underlined), permitting the insertion of the amplified gene into the pET21b vector (Novagen Inc., Madison, WI, USA). The DNA fragment was then inserted into pET21b to produce the plasmid pET21b-*Pa*DHT for *P*. *aeruginosa* dihydropyrimidinase expression. The expected gene product expressed by pET21b-*Pa*DHT has a C-terminal His tag, useful for purifying the recombinant protein.

### Site-directed mutagenesis

The dihydropyrimidinase mutants (H59A, H61A, K150A, Y155A, H183A, H239A, S289A, D316A, and N337A) were generated according to the manufacturer’s protocol (Stratagene, LaJolla, CA). The presence of the mutation was verified by DNA sequencing in each construct. The oligonucleotide primers for the preparation of mutants are shown in Table 1.

**Table 1:** Primers used for construction of plasmids.

Oligonucleotide	Primer
WT-NdeI-N	CGCGGCATATGTTTGATTTACTCCTGC
WT-XhoI-C	TCGCACTCGAGAAAATCGAAGGCATGT
H59A-N	GGCATCGACCCCGCTACCCACATGCAGTTGCCC
H59A-C	CAACTGCATGTGGGTAGCGGGGTCGATGCCGCC
H61A-N	GACCCCCATACCGCCATGCAGTTGCCCTTCATG
H61A-C	GAAGGGCAACTGCATGGCGGTATGGGGGTCGAT
H183A-N	GCGGTGCCGACGGTGGCCGCGGAGAACGGCGAA
H183A-C	GCCGTTCTCCGCGGCCACCGTCGGCACCGCGCC
H239A-N	CTGTACCTGGTAGCTATTTCCAGCCGCGAGGCG
H239A-C	CTCGCGGCTGGAAATAGCTACCAGGTACAGCGG
K150A-N	GTGAACAGCTTCGCGCACTTCATGGCCTACAAG
K150A-C	GTAGGCCATGAAGTGCGCGAAGCTGTTCACCCC
D316A-N	ACCACCGCCACCGCCCACTGCTGCTTCTGCGCC
D316A-C	GCAGAAGCAGCAGTGGGCGGTGGCGGTGGTATG
Y155A-N	CACTTCATGGCCGCCAAGAACGCCATCATGGCC
Y155A-C	CATGATGGCGTTCTTGGCGGCCATGAAGTGCTT
S289A-N	GGCTACGTGATGGCCCCGCCGTTCCGTCCCGTC
S289A-C	GGGACGGAACGGCGGGGCCATCACGTAGCCGGC
N337A-N	TTCAGCAAGATTCCCGCTGGCACGGCCGGCATC
N337A-C	GCCGGCCGTGCCAGCGGGAATCTTGCTGAAGTC

These plasmids were verified by DNA sequencing. Underlined nucleotides indicate the designated site for mutation or the restriction site.

### Protein expression and purification

Recombinant wild-type and mutant dihydropyrimidinases were expressed and purified using the protocol described previously for hydantoinase [12], allantoinase [11,15], dihydroorotase [11,15], PriB [25,26], DnaB [27], DnaT [28,29], and SSB [30,31]. The protein purified from the soluble supernatant by Ni^2+^-affinity chromatography (HiTrap HP; GE Healthcare Bio-Sciences, Piscataway, NJ, USA) was eluted with Buffer A (20 mM Tris-HCl, 250 mM imidazole, and 0.5 M NaCl, pH 7.9) and dialyzed against a dialysis buffer (20 mM HEPES and 100 mM NaCl, pH 7.0; Buffer B). Protein purity remained >97% as determined by SDS-PAGE.

### Enzyme assay

A rapid spectrophotometric assay was used to determine the enzymatic activity according to a previously described protocol for hydantoinase [12], allantoinase [11,15], dihydroorotase [11,15], and imidase [3,4,5]. Dihydrouracil, 5-propyl-hydantoin, and phthalimide were used as substrates. Unless explicitly stated otherwise, dihydrouracil (2 mM) was used as the substrate in the standard assay of dihydropyrimidinase. Briefly, the decrease in absorbancy at 230, 248, and 298 nm was measured upon hydrolysis of dihydrouracil, 5-propyl-hydantoin, and phthalimide as the substrate at 25°C, respectively. To start the reaction, the purified dihydropyrimidinase (10–70 μg) was added to a 2 mL solution containing the substrate and 100 mM Tris–HCl (pH 8.0). Substrate hydrolysis was monitored with a UV/vis spectrophotometer (Hitachi U 3300, Hitachi High-Technologies, Tokyo, Japan). The extinction coefficient of each substrate was determined experimentally by direct measurement with a spectrophotometer. The extinction coefficients of dihydrouracil, 5-propyl-hydantoin, and phthalimide were 0.683 mM^-1^cm^-1^ at 230 nm, 0.0538 mM^-1^cm^-1^ at 248 nm, and 3.12 mM^-1^cm^-1^ at 298 nm, respectively. The initial rates of change were a function of enzyme concentration within the absorbance range of 0.01–0.18 min^-1^. A unit of activity was defined as the amount of enzyme catalyzing the hydrolysis of 1 μmol substrate/min, and the specific activity was expressed in terms of units of activity per milligram of enzyme. The kinetic parameters *K*_m_ and *V*_max_ were determined from a non-linear plot by fitting the hydrolyzing rate from individual experiments to the Michaelis–Menten equation (Enzyme Kinetics module of Sigma-Plot; Systat Software, Chicago, IL, USA).

### Dissociation constants determined by fluorescence spectrophotometer

The dissociation constants (*K*_d_) of dihydropyrimidinase was determined using the fluorescence quenching method as previously described for allantoinase [15], dihydroorotase [15], and DnaB helicase [27,32]. An aliquot of each compound was added into the solution containing dihydropyrimidinase (0.8 μM), 50 mM HEPES at pH 7.0. The decrease in intrinsic fluorescence of protein was measured at 334.5 nm upon excitation at 279 nm and 25°C with a spectrofluorimeter (Hitachi F-2700; Hitachi High-Technologies, Tokyo, Japan). At least seven data points were used to calculate each *K*_d_. Each data point was an average of 2–3 determinants, and the difference of the determinants was within 10%. The *K*_d_ was obtained by the equation: ΔF = ΔF_max_-*K*_d_(ΔF/[compound]) (Enzyme Kinetics module of Sigma-Plot; Systat Software, Chicago, IL, USA).

### Bioinformatics

The amino acid sequences of 497 sequenced dihydropyrimidinase homologues were aligned using ConSurf [33]. The model of *P*. *aeruginosa* dihydropyrimidinase was built using human dihydropyrimidinase (PDB entry: 2VR2) as a template by SWISS-MODEL (http://swissmodel.expasy.org) [34] and (PS)^2^ (http://140.113.239.111/~ps2v2/docs.php) [35]. The coordinate and topology file of the flavonoids was found in DrugBank (http://www.drugbank.ca/) [36]. Myricetin and dihydromyricetin were computationally docked into the three-dimensional model of dihydropyrimidinase by using PatchDock (http://bioinfo3d.cs.tau.ac.il/PatchDock/) [37]. The dihydrouracil-complexed structure model of *P*. *aeruginosa* dihydropyrimidinase was directly constructed by superimposing the crystal structure of the dihydrouracil-yeast dihydropyrimidinase complex (the coordinate of 2FVK). The structures were visualized by using the program PyMol.

## Results

### Expression and purification of a putative dihydropyrimidinase from *P*. *aeruginosa* PAO1

The gene *PA0441* encoding putative dihydropyrimidinase was PCR-amplified using genomic DNA of *P*. *aeruginosa* PAO1 as a template. The amplified gene was then ligated into the pET21b vector for protein expression. *P*. *aeruginosa* dihydropyrimidinase was hetero-overexpressed in *E*. *coli* and purified from the soluble supernatant using Ni^2+^-affinity chromatography. Pure protein was obtained in this single chromatographic step with an elution of buffer A. Approximately 50 mg of purified protein was extracted from 1 L of a culture of *E*. *coli* cells. The mutant dihydropyrimidinases were also purified according to the same protocol used for the wild-type proteins, and yielded very similar purification results.

### Metal-activated dihydropyrimidinase

The catalytic activity of purified dihydropyrimidinase (without any metal supplement in the culture) was not high, so some metal ions were added to the reaction mixture. Table 2 shows that the addition of 1 mM CoCl_2_, ZnCl_2_, or MnCl_2_ activated dihydropyrimidinase activity, and followed the order Co^2+^ > Zn^2+^ > Mn^2+^; CdCl_2_, NiCl_2_, MgCl_2_, and CaCl_2_ were not useful. We also added 1 mM CoCl_2_, the best supplement, into the bacterial culture for dihydropyrimidinase expression, and the resultant dihydropyrimidinase was purified and analyzed. The specific activity of this dihydropyrimidinase toward dihydrouracil was 5.9 ± 0.4 μmol/mg/min, a value very similar to that of the Co^2+^-activated enzyme (5.8 ± 0.5 μmol/mg/min). Thus, dihydropyrimidinase (1 mM CoCl_2_ supplemented into the bacterial culture) was used for all analyses in this study, unless explicitly stated otherwise.

**Table 2:** Effect of metal ions on the activity of dihydropyrimidinase.

Metal ions added	Specific activity	Fold
(1 mM)	(μmol/mg/min)	
None	0.82 ± 0.06	1.0
CoCl_2_	5.80 ± 0.16	7.1
ZnCl_2_	2.90 ± 0.10	3.5
MnCl_2_	1.72 ± 0.09	2.1
CdCl_2_	0.74 ± 0.04	0.9
NiCl_2_	0.91 ± 0.06	1.1
MgCl_2_	0.82 ± 0.05	1.0
CaCl_2_	0.82 ± 0.05	1.0

The activity of purified dihydropyrimidinase (without any metal supplement in the culture) toward dihydrouracil was analyzed by the standard assay. Metal ions were added to the reaction mixture for dihydropyrimidinase activation.

### Substrate specificity and selectivity of dihydropyrimidinase

This enzyme from microorganisms is commonly known as hydantoinase. Although the gene product of *PA0441* has been described as a dihydropyrimidinase, the substrate specificities of dihydropyrimidinase and hydantoinase may differ. For example, the recombinant hydantoinase from *Agrobacterium radiobacter* prefers 5-leucinyl-hydantoin to phthalimide and dihydrouracil (~two orders of magnitude), as revealed by the catalytic efficiencies [12]. To ensure that the gene product of *PA0441* is suitably identified as a dihydropyrimidinase, we analyzed the substrate specificity of this dihydropyrimidinase toward dihydrouracil (Fig 1B), phthalimide (Fig 1C), 5-propyl-hydantoin (Fig 1D), allantoin, and dihydroorotate, which are typical substrates for dihydropyrimidinase, imidase, hydantoinase, allantoinase, and dihydroorotase, respectively (Table 3). Unlike *A*. *radiobacter* hydantoinase, the catalytic efficiency of this enzyme toward dihydrouracil was higher than that toward phthalimide (fourfold) and 5-propyl-hydantoin (100-fold). Therefore, this bacterial enzyme was suitably identified as a dihydropyrimidinase, not a hydantoinase.

**Table 3:** Apparent Michaelis–Menten constants for dihydropyrimidinase using the substrate of each enzyme in the cyclic amidohydrolase family.

	Dihydropyrimidinase			
Substrate	*V*_max_	*K*_m_	*V*_max_/*K*_m_	Fold
Dihydrouracil	7.6 ± 0.4	0.7 ± 0.1	10.9	1.0
Phthalimide	2.7 ± 0.6	1.0 ± 0.3	2.7	0.25
5-Propyl-hydantoin	1.2 ± 0.1	8.1 ± 1.5	0.15	0.01
Allantoin	Not hydrolyzed			
Dihydroorotate	Not hydrolyzed			

The kinetic parameters *K*m and *V*max were determined by fitting the hydrolyzing rate from individual experiments to the Michaelis–Menten equation, and then the standard errors were given.

### Inhibition of dihydropyrimidinase by the substrate analogs

**Fig 2:**
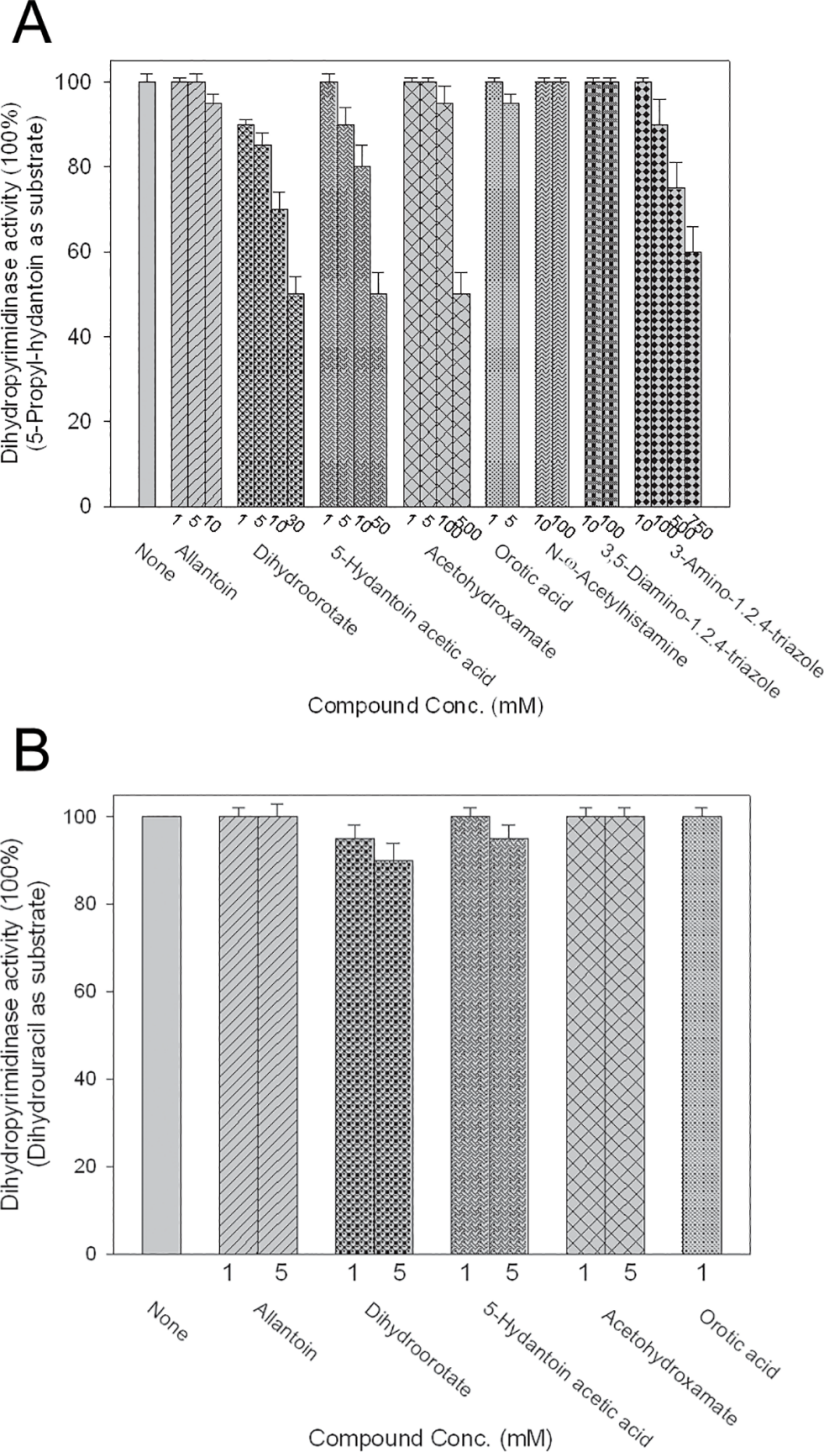
Effect of the substrate and the inhibitor of allantoinase or dihydroorotase on the activity of dihydropyrimidinase using (A) 5-propyl-hydantoin or (B) dihydrouracil as a substrate. Acetohydroxamate and 3-amino-1,2,4-triazole solutions, whose pH values, were pre-adjusted to pH 8. When using dihydrouracil as a substrate, some compounds at high concentrations were difficult to determine the inhibitory effect on the activity of dihydropyrimidinase using the spectrophotometric assay at 230 nm.

Substrate analogs for any enzyme are usually potential inhibitors. Members of the cyclic amidohydrolase family have highly similar active sites, and may use the same catalytic mechanism for substrate hydrolysis [38]. This condition raises an interesting question as to whether the substrate and inhibitor of allantoinase and dihydroorotase can be a potential inhibitor of dihydropyrimidinase because of their structural similarity. Allantoin and dihydroorotate are not substrates for dihydropyrimidinase-catalyzed reactions (Table 3). Allantoin and dihydroorotate are structurally similar to hydantoin and dihydrouracil, except for the 5' side chain (Fig 1A). To assess whether allantoin and dihydroorotate are dihydropyrimidinase inhibitors, allantoin and dihydroorotate were individually included in the standard assay using 5-propyl-hydantoin (Fig 2A) or dihydrouracil (Fig 2B) as a substrate. Allantoin and dihydroorotate only slightly inhibited the activity of dihydropyrimidinase. Thus, substrates of other cyclic amidohydrolase may not be strong inhibitors of dihydropyrimidinase.

To assess whether inhibitors or substrate analogs of allantoinase and dihydroorotase can be inhibitors of dihydropyrimidinase, 5-hydantoin acetic acid, acetohydroxamate, and orotic acid were used in this study. Similarly, 5-hydantoin acetic acid and acetohydroxamate (an inhibitor of allantoinase) could only slightly inhibit the activity of dihydropyrimidinase (Fig 2). We also tested whether inhibitors of the metalloenzyme glutaminyl cyclase, namely, N-ω-acetylhistamine, 3,5-diamino-1,2,4-triazole, and 3-amino-1,2,4-triazole [39,40], are potential inhibitors of dihydropyrimidinase, in which 3-amino-1,2,4-triazole at 100–750 mM affected enzyme activity (Fig 2). High concentrations of these compounds were required to obtain significant inhibition of dihydropyrimidinase. Thus, inhibitors or substrate analogs of allantoinase, dihydroorotase, and glutaminyl cyclase were not potent inhibitors of dihydropyrimidinase.

To compare the inhibitory capability of these compounds on dihydropyrimidinase, their IC_50_ values, the inhibitory concentration required to reduce the activity of the enzyme by 50%, were determined and compared. The IC_50_ values of dihydroorotate, 5-hydantoin acetic acid, and acetohydroxamate were around 30, 50, and 500 mM, respectively. The IC_50_ values of these substrate and inhibitor analogs of other cyclic amidases for dihydropyrimidinase were within the millimolar range.

### Use of flavonoids in dihydropyrimidinase inhibition

**Fig 3:**
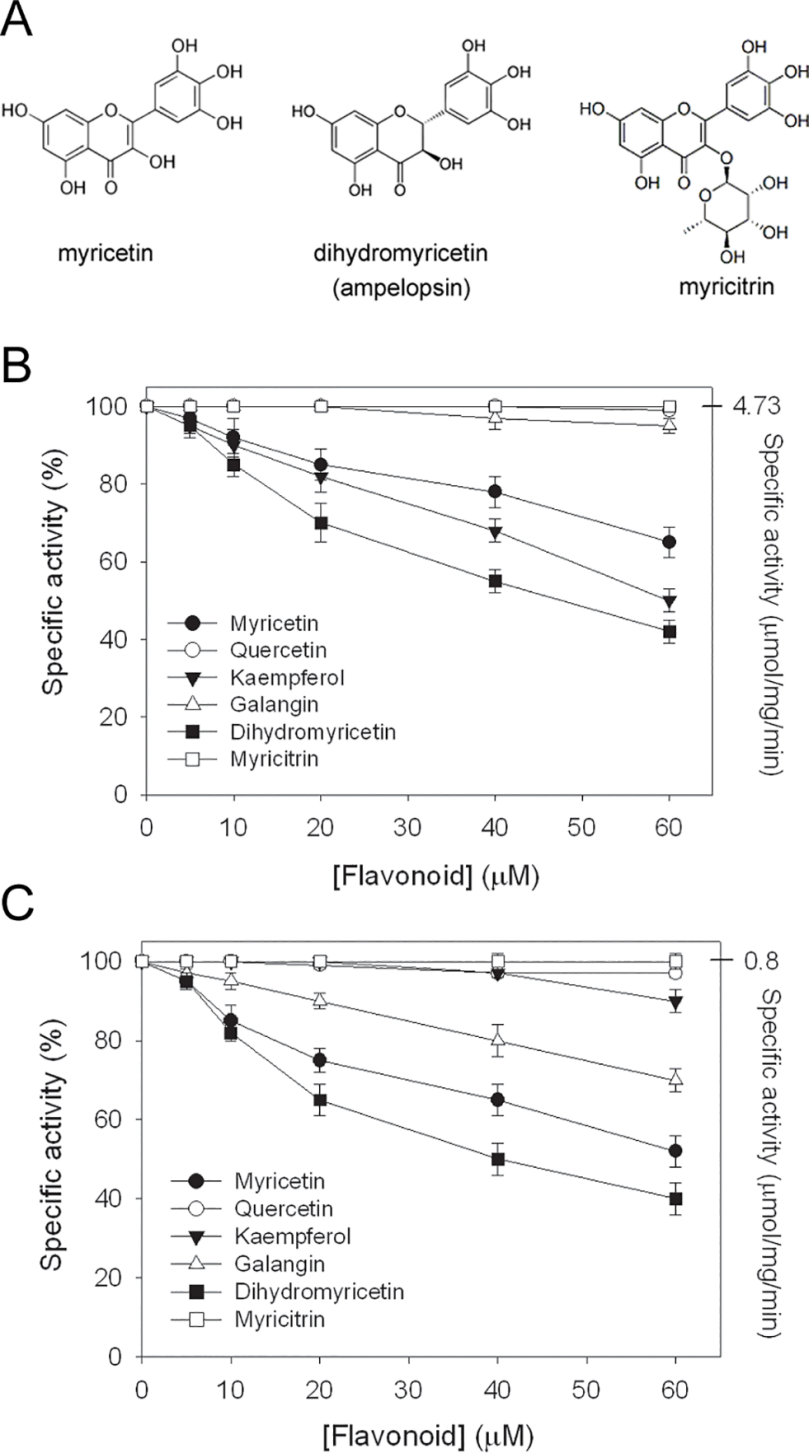
Inhibition of dihydropyrimidinase by flavonoids. (A) Molecular structure of myricetin, dihydromyricetin, and myricitrin. (B) IC_50_ determination of flavonoids for dihydropyrimidinase using dihydrouracil as a substrate. (C) IC_50_ determination of flavonoids for dihydropyrimidinase using 5-propyl-hydantoin as a substrate. IC_50_ value for dihydropyrimidinase was directly determined using graphic analysis.

Substrate analogs for any enzyme are usually potential inhibitors, but this common assumption was not present in this study of dihydropyrimidinase. Given broad substrate specificity, the active site of dihydropyrimidinase can accommodate many different substrates. Therefore, substrate analogs cannot be specifically recognized and cannot significantly inhibit its activity. Although we found that dihydroorotate, 5-hydantoin acetic acid, and acetohydroxamate inhibited dihydropyrimidinase activity, their IC_50_ values were at the millimolar range and higher than the *K*_m_ values of dihydropyrimidinase, which were insufficient as potent inhibitors. To determine whether a naturally occurring product is an inhibitor of dihydropyrimidinase, the inhibitory capabilities of the flavonols myricetin (with three hydroxyl substituents on the aromatic ring), quercetin (with two hydroxyl substituents on the aromatic ring), kaempferol (with one hydroxyl substituent on the aromatic ring), and galangin (without hydroxyl substituent on the aromatic ring) were tested. Furthermore, to study the structure–inhibition relationship with dihydropyrimidinase, the derivatives of myricetin, namely, dihydromyricetin (the ring does not contain a double bond but has additional two hydrogen atoms) and myricitrin (the ring is fused to an additional sugar block), were further used and tested (Fig 3A).

### Identification of flavonol inhibition of dihydropyrimidinase

Flavonols are known to have antioxidant [20], antiradical [22], and antibacterial properties [23,24]. Myricetin, quercetin, kaempferol, and galangin at different concentrations were included in the standard assay, and their IC_50_ values were determined and compared to determine whether flavonols inhibit dihydropyrimidinase. Generally, the activities of dihydropyrimidinase toward dihydrouracil (Fig 3B) and 5-propyl-hydantoin (Fig 3C) continued to decrease as the concentrations of the compound increased. However, these compounds caused distinct effects when different substrates were used. When dihydrouracil was used as a substrate, the inhibitory effects of myricetin and kaempferol on the activity of dihydropyrimidinase were significant; however, kaempferol only slightly inhibited the activity of dihydropyrimidinase toward 5-propyl-hydantoin (Fig 3C). The effect of quercetin was insignificant. Overall, myricetin was found to strongly inhibit the hydrolysis of both dihydrouracil and 5-propyl-hydantoin catalyzed by dihydropyrimidinase.

We further tested whether dihydromyricetin and myricitrin (Fig 3A), derivatives of myricetin, can be inhibitors of dihydropyrimidinase. No effect was found when myricitrin was added into the standard assay using dihydrouracil and 5-propyl-hydantoin as substrates of dihydropyrimidinase. However, opposite to myricitrin, dihydromyricetin exhibited a significant inhibitory effect on the activities of dihydropyrimidinase for both substrates, even more than myricetin did (Fig 3B and 3C). The IC_50_ values of dihydromyricetin for dihydropyrimidinase determined from the titration curves using dihydrouracil and 5-propyl-hydantoin were 48 ± 2 and 40 ± 2 μM, respectively; myricetin yielded higher values of 83 ± 3 and 63 ± 3 μM, respectively. Based on the IC_50_ values, the order of the inhibitory capability of the flavonoids for dihydrouracil was as follows: dihydromyricetin > kaempferol > myricetin > galangin > quercetin > myricitrin. Moreover, the order of the inhibitory capability of the flavonoids for 5-propyl-hydantoin was as follows: dihydromyricetin > myricetin > galangin > kaempferol > quercetin > myricitrin. Thus, for the first time, flavonols, especially dihydromyricetin, were established as novel inhibitors of dihydropyrimidinase. In addition, the inhibitory capabilities of these flavonols (at the μM range) were significantly higher than those of the substrate and inhibitor analogs (at the mM range) of dihydropyrimidinase.

### Structural modeling of dihydropyrimidinase

To study the structure–inhibition relationship between dihydropyrimidinase and the flavonoids in silico, the structure of dihydropyrimidinase was modeled and used for docking experiments. The crystal structure of *P*. *aeruginosa* dihydropyrimidinase has yet to be determined. We modeled the *P*. *aeruginosa* dihydropyrimidinase structure (S1 Fig) by homology modeling using SWISS-MODEL (http://swissmodel.expasy.org/) [34]. Human dihydropyrimidinase (PDB entry: 2VR2) was the first hit suggested as a template by the program. The amino acid sequences of human (with 519 aa) and *P*. *aeruginosa* dihydropyrimidinase (with 479 aa) shared 51% identity and 66% similarity (S2 Fig). We also used (PS)^2^, another bioinformatic tool, for structural modeling [35]. (PS)^2^ (http://140.113.239.111/~ps2v2/docs.php) is an automatic homology modeling server that combines both sequence and secondary structure information to detect the homologous proteins with remote similarity and target–template alignment. Human dihydropyrimidinase was also the first hit suggested as a template by (PS)^2^ because it had the highest score. Results obtained from (PS)^2^ analysis indicated that 99.58% of the secondary structure was aligned, which implied that human and *P*. *aeruginosa* dihydropyrimidinase shared a highly similar structure (S3 Fig).

### Mutational analysis of the residues within the active site

**Fig 4:**
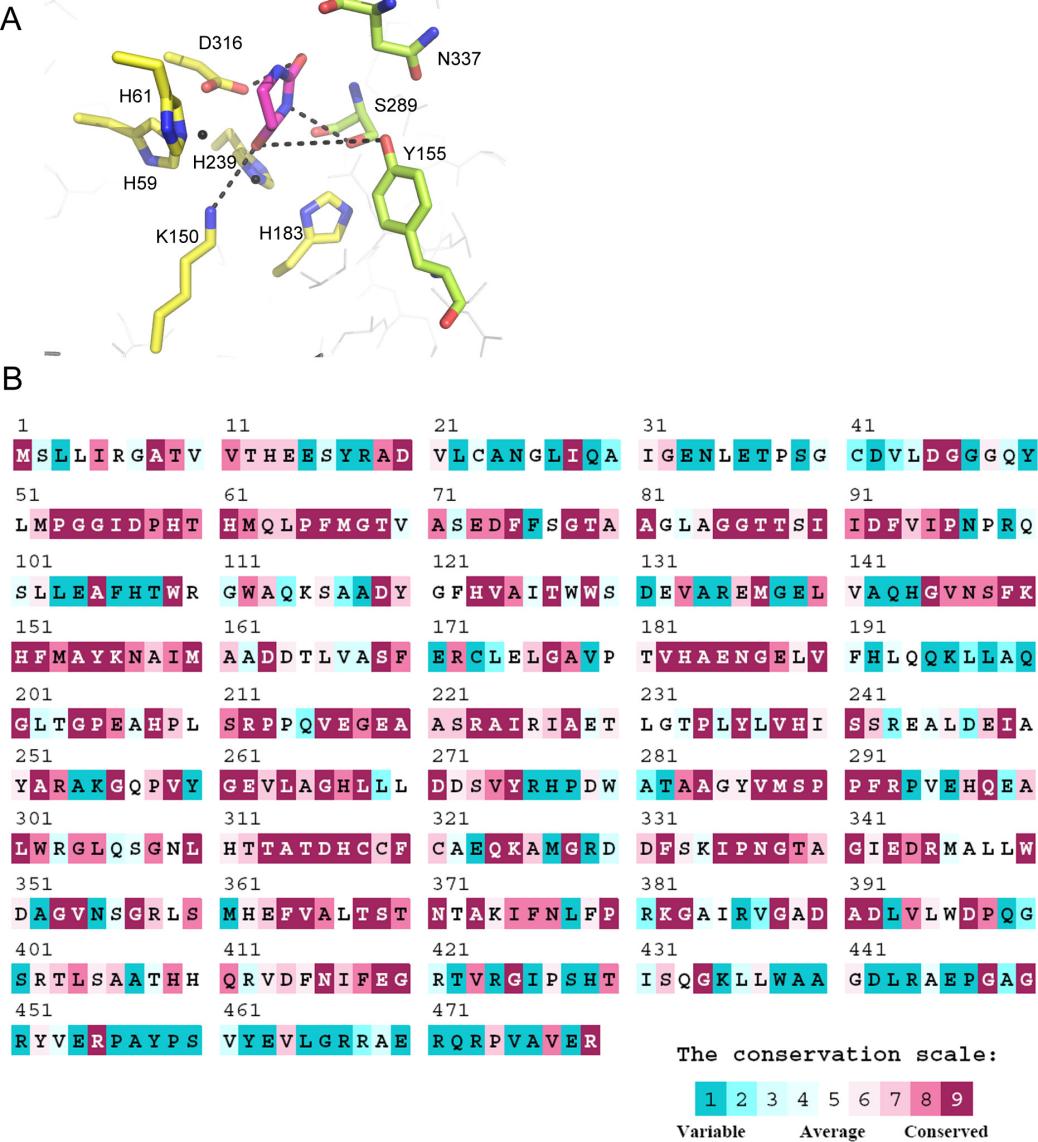
The active site of dihydropyrimidinase. (A) The active site of *P*. *aeruginosa* dihydropyrimidinase. According to the crystal structure of *Saccharomyces kluyveri* (PDB entry: 2FVK), residues H59, H61, K150, H183, H239, and D316 of *P*. *aeruginosa* dihydropyrimidinase shown in yellow were crucial for the assembly of the binuclear metal center within the active site; meanwhile, residues Y155, S289, and N337 shown in limon were crucial for substrate binding. The model was directly constructed by superimposing the modeled structure of *P*. *aeruginosa* dihydropyrimidinase with the crystal structure of *S*. *kluyveri* dihydropyrimidinase-dihydrouracil complex. Dihydrouracil generated from the complex is shown in light magenta. (B) An alignment consensus of 497 sequenced dihydropyrimidinase homologs by ConSurf reveals the degree of variability at each position along the primary sequence. Note that the positions involved in the assembly of the binuclear metal center within the active site and the substrate binding of *P*. *aeruginosa* dihydropyrimidinase are well conserved.

According to the crystal structure of *Saccharomyces kluyveri* (PDB entry: 2FVK) [41] and *Sinorhizobium meliloti* dihydropyrimidinases (PDB entry: 3DC8) [42], residues H59, H61, K150, H183, H239, and D316 of *P*. *aeruginosa* dihydropyrimidinase were crucial for the assembly of the binuclear metal center within the active site; meanwhile, residues Y155, S289, and N337 were crucial for substrate binding (Fig 4A). The amino acid sequences of 497 sequenced dihydropyrimidinase homologs aligned using ConSurf [33] indicated that these residues were well conserved (Fig 4B). The importance of these residues was then probed by site-directed mutagenesis, in which alanine substitution was constructed and analyzed. As expected, the catalytic activities of these Ala-substituted mutant proteins were severely impaired. H59A, H61A, K150A, H183A, H239A, D316A, Y155A and S289A were inactive. Only N337A mutant protein was found to be active, but its activity was about 20-fold less than that of the wild-type dihydropyrimidinase.

### Structural models of the binding mode of dihydropyrimidinase to dihydromyricetin and myricetin

**Fig 5:**
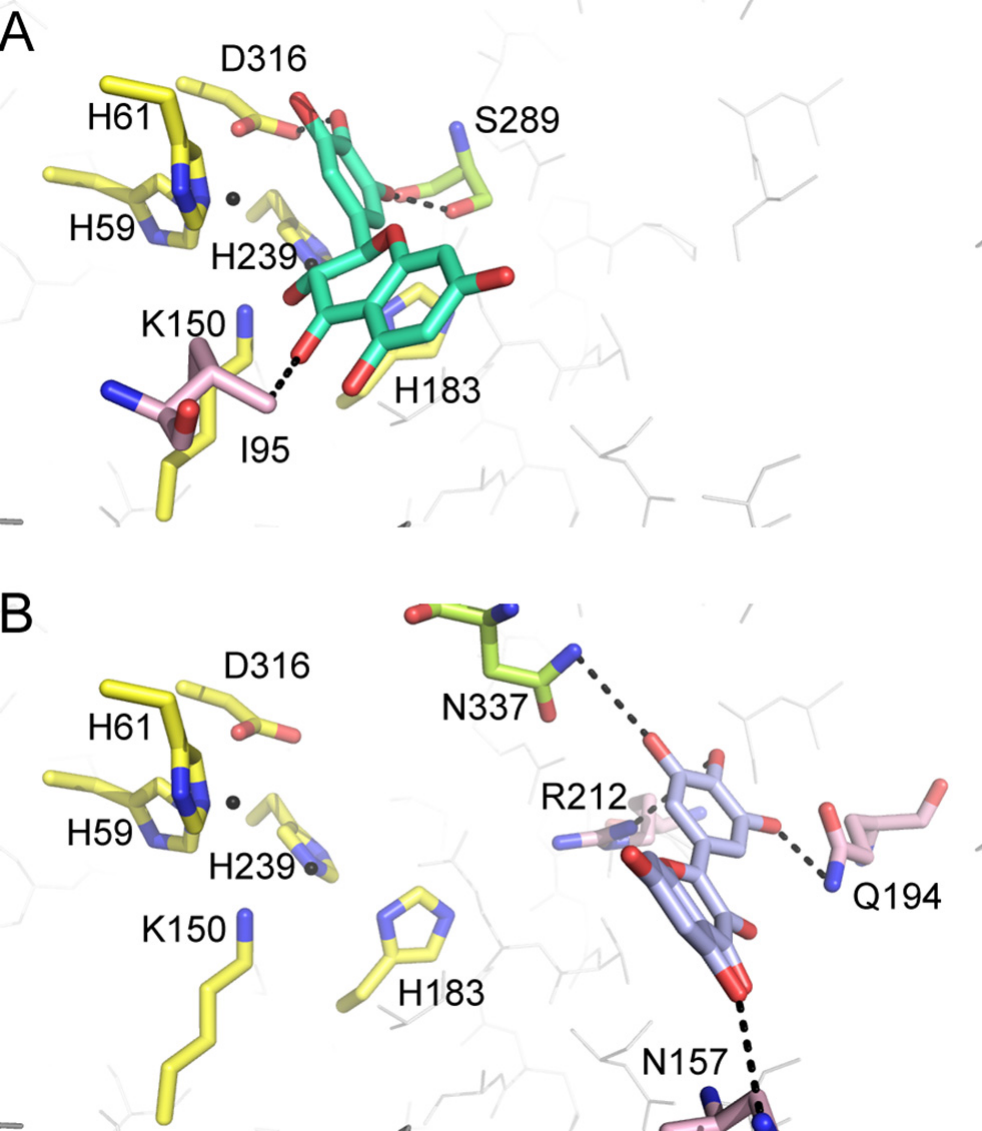
Representation of the docking models of the dihydropyrimidinase complex from PatchDock. (A) The binding mode of dihydropyrimidinase to dihydromyricetin. Dihydromyricetin interacted with I95 (light pink), S289 (limon), and D316 (yellow) of dihydropyrimidinase, in which S289 and D316 were found to be crucial for the catalytic activity of dihydropyrimidinase. (B) The binding mode of dihydropyrimidinase to myricetin. Myricetin interacted with N157 (light pink), Q194 (light pink), R212 (light pink), and N337 (limon), in which N337 was found to be crucial for the catalytic activity of dihydropyrimidinase.

To understand the inhibitory mechanism of dihydromyricetin and myricetin on dihydropyrimidinase, their structures found in the DrugBank (http://www.drugbank.ca/) [36] were computationally docked into the 3D model of *P*. *aeruginosa* dihydropyrimidinase using PatchDock (http://bioinfo3d.cs.tau.acil/PatchDock/) [37]. Docking was automatically performed after uploading the coordinates and topology files of the compound and dihydropyrimidinase. The docking models with the highest score in dihydropyrimidinase interacting with dihydromyricetin and myricetin are shown in Fig 5. Although dihydromyricetin and myricetin were docked into the active site pocket of dihydropyrimidinase, their binding modes differed. On one hand, dihydromyricetin interacted with I95, S289, and D316 (Fig 5A), in which S289 and D316 were found to be crucial for the catalytic activity of dihydropyrimidinase. On the other hand, myricetin interacted with N157, Q194, R212, and N337, in which only N337 was crucial. This docking study showed that myricetin only partially occupied the dihydropyrimidinase active site. The ring structure of dihydromyricetin was more flexible than that of myricetin (Fig 5) because of lacking the double bond on its ring (Fig 3A).

### Dihydromyricetin is a competitive inhibitor of dihydropyrimidinase

**Fig 6:**
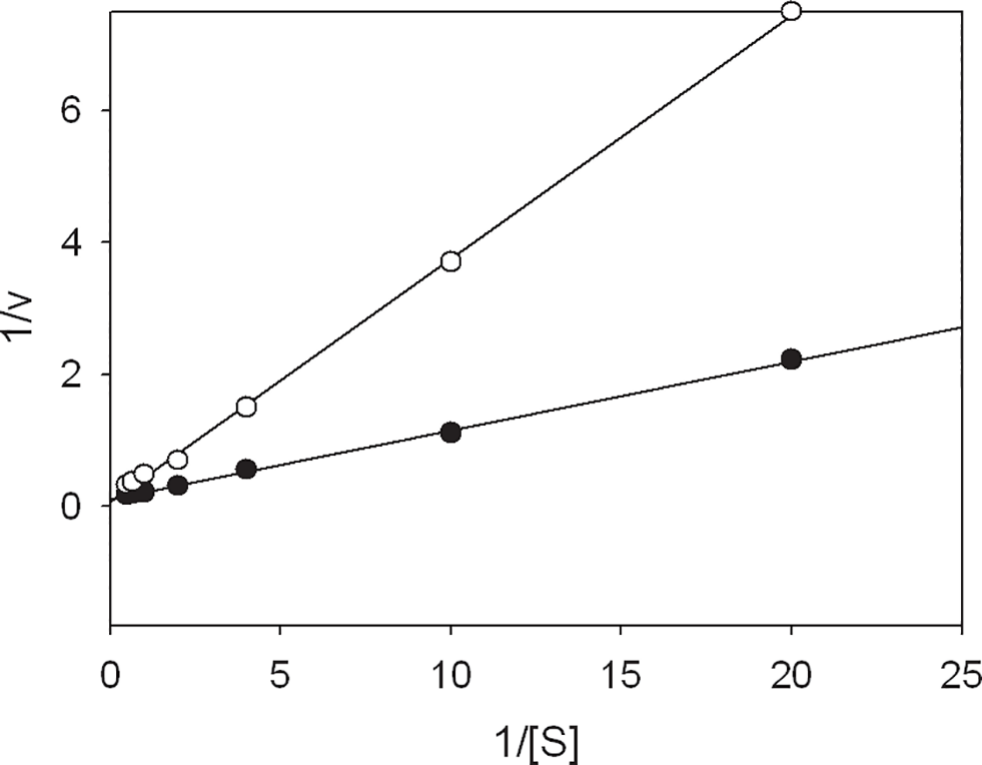
Dihydromyricetin is a competitive inhibitor for dihydropyrimidinase. Kinetic study of dihydropyrimidinase with (open circles) and without dihydromyricetin (close circles). Inhibition of dihydropyrimidinase by dihydromyricetin (40 μM) resulted in Lineweaver–Burk plots where the lines are cross the y-axis at the similar point, indicating that dihydromyricetin is a competitive inhibitor for dihydropyrimidinase. Data points are an average of 2–3 determinations within 10% error.

We identified dihydromyricetin as a potent inhibitor with an IC_50_ value of 48 μM on dihydropyrimidinase. To determine what type of inhibitor dihydromyricetin is, this compound (40 μM) was included in the standard assay for dihydropyrimidinase with different concentrations of dihydrouracil. As shown in Fig 6, inhibition of dihydropyrimidinase by dihydromyricetin resulted in Lineweaver−Burk plots with lines that crossed the y-axis at a similar point, which indicated that dihydromyricetin was a competitive inhibitor of dihydropyrimidinase. The presence of 40 μM dihydromyricetin yielded *V*_max_ and *K*_m_ values, kinetic constants, of 5.4 ± 0.4 μmol/mg/min and 1.6 ± 0.2 mM, respectively. Without dihydromyricetin, the kinetic constants *V*_max_ and *K*_m_ were 7.6 ± 0.4 μmol/mg/min and 0.7 ± 0.1 mM, respectively (Table 3). The *K*_m_ value obviously increased by twofold, whereas *V*_max_ was only slightly affected. These findings indicated the competitive inhibition of dihydropyrimidinase by dihydromyricetin; specifically, dihydromyricetin could compete with dihydrouracil for the active site of dihydropyrimidinase [43].

### *K*_d_ values of dihydropyrimidinase bound to dihydromyricetin and myricetin

**Fig 7:**
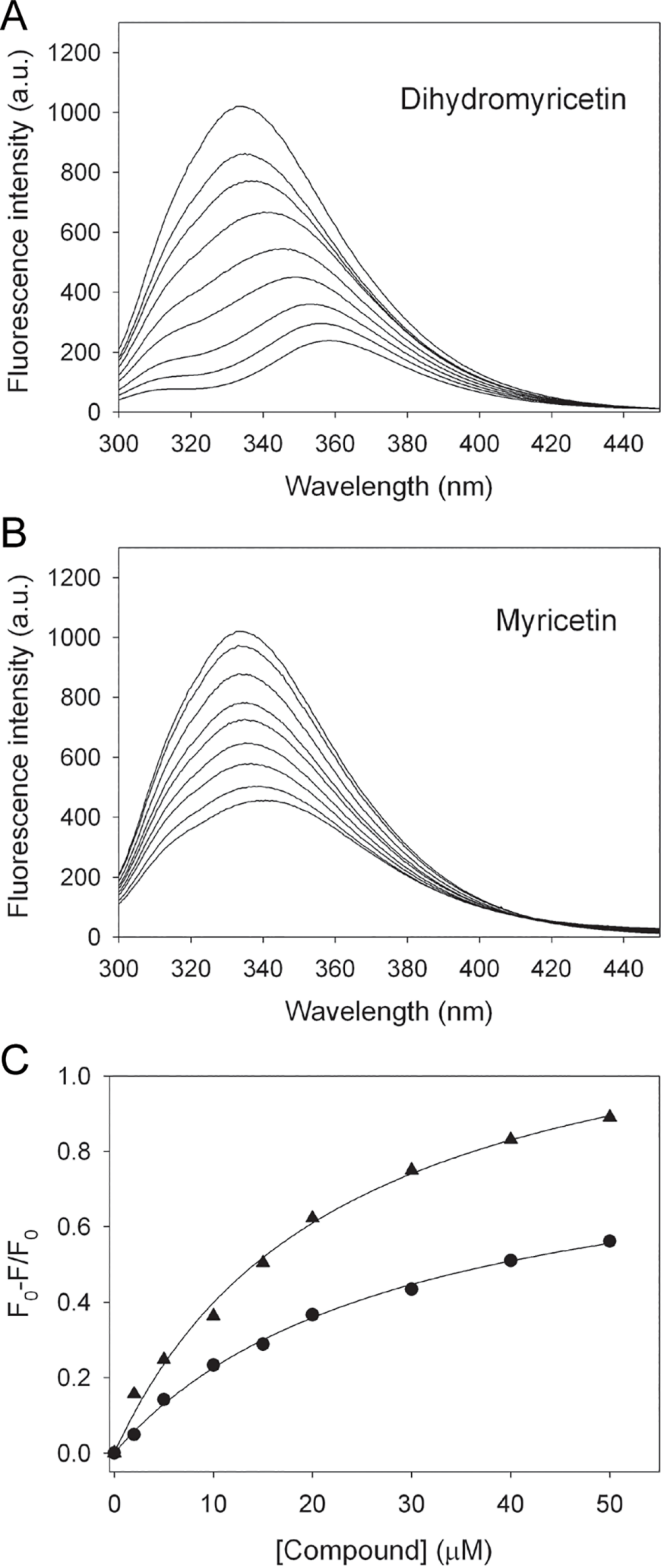
The fluorescence emission spectra of dihydropyrimidinase. The decrease in intrinsic fluorescence of protein was measured at 334.5 nm upon excitation at 279 nm and 25°C with a spectrofluorimeter. (A) The fluorescence emission spectra of dihydropyrimidinase with dihydromyricetin of different concentrations (0–50 μM). The fluorescence intensity emission spectra of dihydropyrimidinase significantly quenched with dihydromyricetin. (B) The fluorescence emission spectra of dihydropyrimidinase with myricetin of different concentrations (0–50 μM). The fluorescence intensity emission spectra of dihydropyrimidinase significantly quenched with myricetin. (C) Fluorescence titrations of dihydromyricetin and myricetin with dihydropyrimidinase. An aliquot amount of dihydromyricetin and myricetin was individually added to the enzyme solution for each *K*_d_. The *K*_d_ was obtained by the equation: ΔF = ΔF_max_-*K*_d_(ΔF/[compound]). Data points are an average of 2–3 determinations within 10% error.

To determine whether the inhibitory capabilities of myricetin and dihydromyricetin are correlated with their binding abilities, the dissociation constants (the *K*_d_ values) of dihydropyrimidinase for myricetin and dihydromyricetin were determined through fluorescence quenching. Quenching refers to the complex formation process that decreases the fluorescence intensity of the protein. The fluorescence emission spectra of dihydropyrimidinase were remarkably quenched with dihydromyricetin (Fig 7A) and myricetin (Fig 7B). Upon adding 50 μM myricetin and dihydromyricetin, the intrinsic fluorescence of dihydropyrimidinase was quenched by 56.2% and 89.1%, respectively. As shown in Fig 7B, adding different concentrations of myricetin resulted in a red shift (~7 nm; λ_max_ = 333.5–340.5 nm) in the dihydropyrimidinase emission wavelength (λ_em_). Adding dihydromyricetin also caused a red shift in the dihydropyrimidinase emission wavelength (Fig 7A), which was much more significant (~25 nm; λ_max_ = 333.5–358.5 nm). These observations indicated that myricetin and dihydromyricetin interacted with dihydropyrimidinase, thereby suggesting that myricetin and dihydromyricetin could form stable complexes with dihydropyrimidinase. The *K*_d_ values of dihydropyrimidinase bound to myricetin and dihydromyricetin, as determined through their titration curves (Fig 7C), were 28.8 ± 2.2 and 22.6 ± 2.6 μM, respectively.

### *K*_d_ values of the N337A mutant bound to dihydromyricetin and myricetin

**Fig 8:**
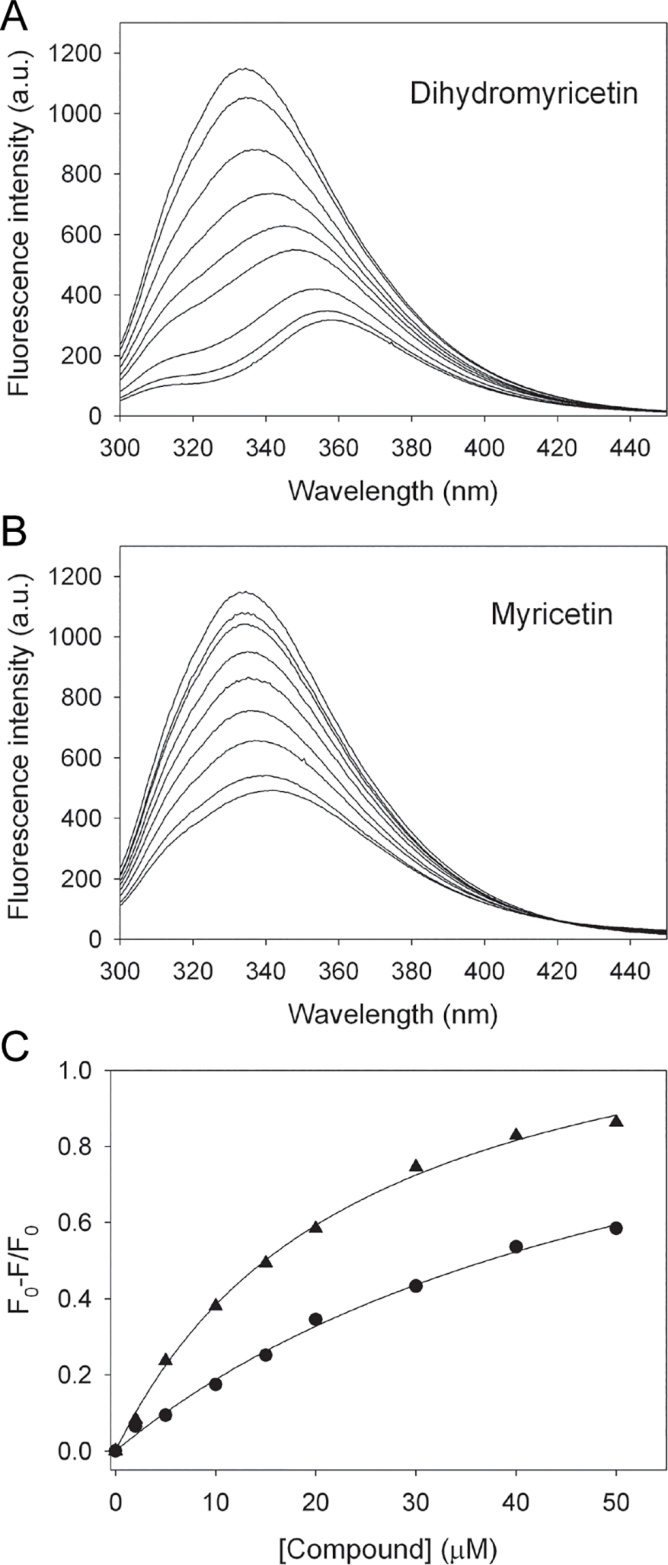
The fluorescence emission spectra of the N337A mutant. (A) The fluorescence emission spectra of the N337A mutant with dihydromyricetin of different concentrations (0–50 μM). (B) The fluorescence emission spectra of the N337A mutant with myricetin of different concentrations (0–50 μM). (C) Fluorescence titrations of dihydromyricetin and myricetin with the N337A mutant. An aliquot amount of dihydromyricetin and myricetin was individually added to the enzyme solution for each *K*_d_. The *K*_d_ was obtained by the equation: ΔF = ΔF_max_-*K*_d_(ΔF/[compound]). Data points are an average of 2–3 determinations within 10% error.

Our in silico experiments showed that myricetin and dihydromyricetin may use different modes to bind to dihydropyrimidinase (Fig 5). These different possible binding modes were also revealed by the distinct fluorescence quenching spectra, in which their λ_max_ shifts differed significantly (Fig 7). The docking study showed that N337 interacted with the hydroxyl group on the ring of myricetin, but not dihydromyricetin. Thus, fluorescence quenching of the N337A mutant was also carried out to test whether this residue is important for myricetin binding, but not for dihydromyricetin. Myricetin and dihydromyricetin quenched the intrinsic fluorescence of dihydropyrimidinase by 58.4% and 86.3%, respectively (Fig 8). Adding myricetin and dihydromyricetin resulted in red shifts in λ_max_ of the N337A mutant from 333.5 nm to 341 nm (~7.5 nm) and 357 nm (~23.5 nm), respectively. The λ_max_ shifts of the N337A mutant by myricetin and dihydromyricetin were similar, but still slightly different, to those of the wild-type dihydropyrimidinase (Fig 7). The *K*_d_ value of the N337A mutant bound to myricetin (Fig 8C), compared with that of the wild-type dihydropyrimidinase, was reduced to 59.3 ± 8.6 μM (twofold). However, the *K*_d_ value of the N337A mutant for dihydromyricetin binding was 24.1 ± 1.7 μM, a value nearly identical to that of the wild-type dihydropyrimidinase, which suggested that N337 was important for myricetin binding but not for dihydromyricetin. These observations indicated that myricetin and dihydromyricetin could still form stable complexes with the N337A mutant, but the binding environment of the active site within the N337A mutant was somewhat different from that of the wild-type protein.

## Discussion

Substrate analogs for any enzyme are usually potential inhibitors, but this common rule is not applicable for dihydropyrimidinase (Fig 2). Combating diseases caused by infections resistant to all antibacterial options requires the development of clinically useful small-molecule antibiotics and identification of novel targets. DNA metabolism is one of the most basic biological functions that should be a prime target in antibiotic development. Considering that dihydropyrimidinase is a component in the chain of pyrimidine catabolism required for metabolizing the DNA base, blocking this enzyme’s activity may be useful to limit bacterial growth and survival. Although some chelators are known to inhibit dihydropyrimidinase, they are harmful to humans. In this study, we showed that dihydromyricetin, a flavonol, significantly inhibited the catalytic activities of dihydropyrimidinase toward both the natural substrate dihydrouracil and xenobiotic substrate 5-propyl-hydantoin (Fig 3). Furthermore, the metabolic effects and safety of the flavonols are well established, making flavonols beneficial for humans. Thus, some of these plant polyphenols may become the lead compounds for further antibiotic development and drug design.

We found that the flavonols myricetin, quercetin, kaempferol, and galangin, which contain different numbers of hydroxyl substituents on their aromatic rings, exerted different inhibitory effects on dihydropyrimidinase (Fig 3). Although flavonols are known to have several hydroxyl groups, thought to have remarkable potential for binding any protein, the strength of the flavonols’ inhibitory effect (IC_50_) on dihydropyrimidinase was not correlated with the number of hydroxyl substituents on the flavonols’ aromatic rings. In addition, the inhibitory effect (IC_50_) of flavonols on dihydropyrimidinase was substrate-dependent. For example, as shown in Fig 3, the inhibitory effect of kaempferol on the activity of dihydropyrimidinase was significant only with dihydrouracil as a substrate (with IC_50_ value of 50 ± 2 μM), but not with 5-propyl-hydantoin. Although our docking results (Fig 5) and the findings of the kinetic studies (Fig 6) both showed a competitive inhibition of the flavonol dihydromyricetin on dihydropyrimidinase, further structure–inhibition relationship studies are still needed for deeper understanding of the inhibition mechanism, particularly because we are still unsure whether each enzyme only contains a single dihydromyricetin within the active site of dihydropyrimidinase.

Flavonoids are the most common group of plant polyphenols with antioxidant, antiradical, anticancer, and antibacterial properties [19]. Some flavonoids are ATPase-inhibiting agents and competitors for ATP-binding proteins [27,32]. For example, several flavonoid derivatives have been developed as therapeutic agents for cancer [21]. Myricetin and scutellarein strongly inhibit the ATPase activity of nsP13 of the SARS coronavirus helicase [44]. Myricetin, luteolin, and morin inhibit the hexameric replicative helicases, and myricetin inhibits Gram-negative bacteria growth [45]. Myricetin also inhibits bacterial allantoinase and dihydroorotase [15]. Given that myricetin is also a potent inhibitor of numerous DNA and RNA polymerases and telomerases [46], these flavonoids, especially myricetin, may be a competent dirty drug or multi-target drug [43].

Dihydropyrimidinase is a zinc enzyme. However, in this study, dihydropyrimidinase supplemented with Co^2+^ ions exhibited the highest activity (Table 2). Due to the low bioavailability of cobalt on earth and in cells, we thought that the possibility of a biological role playing in dihydropyrimidinase may be ruled out. The higher activity of the Co^2+^-substituted enzymes has been proposed. The higher activity followed by metal replacement in Co(II)-thermolysin than in native thermolysin may be due to the enhancement of stabilization of the transition state than those of the native thermolysin [47]. For imidase, the sizes of the metal are found as the important factors that affect the activity of imidase [4]. The position of nucleophilic attack on an imide substrate may fluctuate due to size of the coordinating metal ion. Because the crystal structure of the Co^2+^-dihydropyrimidinase is still lacking, both these possibilities cannot be ruled out.

The chemical mechanism of the binuclear metal center-containing amidohydrolase likely involves three steps: (1) the hydrolytic water molecule must be activated for nucleophilic attack, (2) the amide bond of the substrate must be made more electrophilic by the polarization of the carbonyl–oxygen bond, and (3) the leaving group nitrogen must be protonated as the carbon-nitrogen bond is cleaved [38,48]. This catalytic mechanism is applicable to each member in the cyclic amidohydrolase family [41,48,49]. However, a question remains as to why a common mechanism-based inhibitor for these imide-hydrolyzing enzymes, such as imidase, hydantoinase, dihydropyrimidinase, allantoinase, and dihydroorotase, is difficult to find. New and undetermined factors should be further studied and discovered in this protein family, such as the third metal ion recently found in dihydroorotase, which was found to be highly important for catalysis [50,51]. Further studies are also necessary to investigate the substrate specificity, selectivity, and catalytic mechanism of dihydropyrimidinase and hydantoinase, as well as other cyclic amidohydrolases.

## Supporting Information

S1 Fig Structure modeling of *P*. *aeruginosa* dihydropyrimidinase by SWISS-MODEL. *P*. *aeruginosa* dihydropyrimidinase structure was modeled using SWISS-MODEL and human dihydropyrimidinase (PDB entry: 2VR2) as a template. The amino acid residue 3−479 in the modeled structure of *P*. *aeruginosa* dihydropyrimidinase (limon) was superimposed with the amino acid residue 5−493 in the crystal structure of human dihydropyrimidinase (gray). A tetrameric structure of human dihydropyrimidinase was shown.
(TIF)

S2 Fig The sequence alignment. The amino acid sequence of *P*. *aeruginosa* dihydropyrimidinase (Query) was aligned with human dihydropyrimidinase (Sbjct). The amino acid sequences of human (with 519 aa) and *P*. *aeruginosa* dihydropyrimidinase (with 479 aa) share 51% identity and 66% similarity.
(TIF)

S3 Fig Structure analysis of *P*. *aeruginosa* dihydropyrimidinase by (PS)^2^. Result from (PS)^2^ analysis showed that 99.58% of the secondary structure is aligned, indicating a highly similar structure between human and *P*. *aeruginosa* dihydropyrimidinase.
(TIF)

## References

[1] Schnackerz KD, Dobritzsch D (2008) Amidohydrolases of the reductive pyrimidine catabolic pathway purification, characterization, structure, reaction mechanisms and enzyme deficiency. Biochim Biophys Acta 1784: 431–444. 10.1016/j.bbapap.2008.01.005 18261476

[2] Wallach DP, Grisolia S (1957) The purification and properties of hydropyrimidine hydrase. J Biol Chem 226: 277–288. 13428761

[3] Huang CY, Yang YS (2003) A novel cold-adapted imidase from fish *Oreochromis niloticus* that catalyzes hydrolysis of maleimide. Biochem Biophys Res Commun 312: 467–472. 14637160 10.1016/j.bbrc.2003.10.151

[4] Huang CY, Yang YS (2002) The role of metal on imide hydrolysis: metal content and pH profiles of metal ion-replaced mammalian imidase. Biochem Biophys Res Commun 297: 1027–1032. 12359259 10.1016/s0006-291x(02)02330-6

[5] Yang YS, Ramaswamy S, Jakoby WB (1993) Rat liver imidase. J Biol Chem 268: 10870–10875. 8388376

[6] Yang YL, Ramaswamy SG, Jakoby WB (1998) Enzymatic hydrolysis of organic cyclic carbonates. J Biol Chem 273: 7814–7817. 9525873 10.1074/jbc.273.14.7814

[7] Schoemaker HE, Mink D, Wubbolts MG (2003) Dispelling the myths—biocatalysis in industrial synthesis. Science 299: 1694–1697. 12637735 10.1126/science.1079237

[8] Altenbuchner J, Siemann-Herzberg M, Syldatk C (2001) Hydantoinases and related enzymes as biocatalysts for the synthesis of unnatural chiral amino acids. Curr Opin Biotechnol 12: 559–563. 11849938 10.1016/s0958-1669(01)00263-4

[9] Hsu CC, Lu LY, Yang YS (2010) From sequence and structure of sulfotransferases and dihydropyrimidinases to an understanding of their mechanisms of action and function. Expert Opin Drug Metab Toxicol 6: 591–601. 10.1517/17425251003601987 20397966

[10] Holm L, Sander C (1997) An evolutionary treasure: unification of a broad set of amidohydrolases related to urease. Proteins 28: 72–82. 9144792

[11] Ho YY, Huang YH, Huang CY (2013) Chemical rescue of the post-translationally carboxylated lysine mutant of allantoinase and dihydroorotase by metal ions and short-chain carboxylic acids. Amino Acids 44: 1181–1191. 10.1007/s00726-012-1451-3 23287969

[12] Huang CY, Hsu CC, Chen MC, Yang YS (2009) Effect of metal binding and posttranslational lysine carboxylation on the activity of recombinant hydantoinase. J Biol Inorg Chem 14: 111–121. 10.1007/s00775-008-0428-x 18781344

[13] Evans DR, Guy HI (2004) Mammalian pyrimidine biosynthesis: fresh insights into an ancient pathway. J Biol Chem 279: 33035–33038. 15096496 10.1074/jbc.R400007200

[14] Kumar V, Saxena N, Sarma M, Radha Kishan KV (2011) Carboxylated lysine is required for higher activities in hydantoinases. Protein Pept Lett 18: 663–669. 21413921 10.2174/092986611795446049

[15] Peng WF, Huang CY (2014) Allantoinase and dihydroorotase binding and inhibition by flavonols and the substrates of cyclic amidohydrolases. Biochimie 101: 113–122. 10.1016/j.biochi.2014.01.001 24418229

[16] Bush K (2010) Alarming beta-lactamase-mediated resistance in multidrug-resistant *Enterobacteriaceae* . Curr Opin Microbiol 13: 558–564. 10.1016/j.mib.2010.09.006 20920882

[17] Zhao WH, Hu ZQ (2010) Beta-lactamases identified in clinical isolates of *Pseudomonas aeruginosa* . Crit Rev Microbiol 36: 245–258. 10.3109/1040841X.2010.481763 20482453

[18] Koul A, Arnoult E, Lounis N, Guillemont J, Andries K (2011) The challenge of new drug discovery for tuberculosis. Nature 469: 483–490. 10.1038/nature09657 21270886

[19] Ross JA, Kasum CM (2002) Dietary flavonoids: bioavailability, metabolic effects, and safety. Annu Rev Nutr 22: 19–34. 12055336 10.1146/annurev.nutr.22.111401.144957

[20] Wolfe KL, Liu RH (2008) Structure-activity relationships of flavonoids in the cellular antioxidant activity assay. J Agric Food Chem 56: 8404–8411. 10.1021/jf8013074 18702468

[21] Teillet F, Boumendjel A, Boutonnat J, Ronot X (2008) Flavonoids as RTK inhibitors and potential anticancer agents. Med Res Rev 28: 715–745. 18080331 10.1002/med.20122

[22] Burda S, Oleszek W (2001) Antioxidant and antiradical activities of flavonoids. J Agric Food Chem 49: 2774–2779. 11409965 10.1021/jf001413m

[23] Daglia M (2012) Polyphenols as antimicrobial agents. Curr Opin Biotechnol 23: 174–181. 10.1016/j.copbio.2011.08.007 21925860

[24] Cushnie TP, Lamb AJ (2005) Antimicrobial activity of flavonoids. Int J Antimicrob Agents 26: 343–356. 16323269 10.1016/j.ijantimicag.2005.09.002 PMC7127073

[25] Huang YH, Lo YH, Huang W, Huang CY (2012) Crystal structure and DNA-binding mode of *Klebsiella pneumoniae* primosomal PriB protein. Genes Cells 17: 837–849. 10.1111/gtc.12001 22938024

[26] Hsieh HC, Huang CY (2011) Identification of a novel protein, PriB, in *Klebsiella pneumoniae* . Biochem Biophys Res Commun 404: 546–551. 10.1016/j.bbrc.2010.12.023 21144832

[27] Lin HH, Huang CY (2012) Characterization of flavonol inhibition of DnaB helicase: real-time monitoring, structural modeling, and proposed mechanism. J Biomed Biotechnol 2012: 735368 10.1155/2012/735368 23091356 PMC3468084

[28] Huang YH, Lin MJ, Huang CY (2013) DnaT is a single-stranded DNA binding protein. Genes Cells 18: 1007–1019. 10.1111/gtc.12095 24118681

[29] Huang YH, Huang CY (2013) The N-terminal domain of DnaT, a primosomal DNA replication protein, is crucial for PriB binding and self-trimerization. Biochem Biophys Res Commun 442: 147–152. 10.1016/j.bbrc.2013.11.069 24280305

[30] Huang YH, Huang CY (2014) C-terminal domain swapping of SSB changes the size of the ssDNA binding site. Biomed Res Int 2014: 573936 10.1155/2014/573936 25162017 PMC4137731

[31] Huang YH, Huang CY (2012) Characterization of a single-stranded DNA-binding protein from *Klebsiella pneumoniae*: mutation at either Arg73 or Ser76 causes a less cooperative complex on DNA. Genes Cells 17: 146–157. 10.1111/j.1365-2443.2011.01577.x 22244199

[32] Chen CC, Huang CY (2011) Inhibition of *Klebsiella pneumoniae* DnaB helicase by the flavonol galangin. Protein J 30: 59–65. 10.1007/s10930-010-9302-0 21210194

[33] Landau M, Mayrose I, Rosenberg Y, Glaser F, Martz E, Pupko T, et al (2005) ConSurf 2005: the projection of evolutionary conservation scores of residues on protein structures. Nucleic Acids Res 33: W299–302. 15980475 10.1093/nar/gki370 PMC1160131

[34] Arnold K, Bordoli L, Kopp J, Schwede T (2006) The SWISS-MODEL workspace: a web-based environment for protein structure homology modelling. Bioinformatics 22: 195–201. 16301204 10.1093/bioinformatics/bti770

[35] Chen CC, Hwang JK, Yang JM (2006) (PS)^2^: protein structure prediction server. Nucleic Acids Res 34: W152–157. 16844981 10.1093/nar/gkl187 PMC1538880

[36] Knox C, Law V, Jewison T, Liu P, Ly S, Frolkis A, et al (2011) DrugBank 3.0: a comprehensive resource for 'omics' research on drugs. Nucleic Acids Res 39: D1035–1041. 10.1093/nar/gkq1126 21059682 PMC3013709

[37] Schneidman-Duhovny D, Inbar Y, Nussinov R, Wolfson HJ (2005) PatchDock and SymmDock: servers for rigid and symmetric docking. Nucleic Acids Res 33: W363–367. 15980490 10.1093/nar/gki481 PMC1160241

[38] Seibert CM, Raushel FM (2005) Structural and catalytic diversity within the amidohydrolase superfamily. Biochemistry 44: 6383–6391. 15850372 10.1021/bi047326v

[39] Huang KF, Hsu HL, Karim S, Wang AH (2014) Structural and functional analyses of a glutaminyl cyclase from Ixodes scapularis reveal metal-independent catalysis and inhibitor binding. Acta Crystallogr D Biol Crystallogr 70: 789–801. 10.1107/S1399004713033488 24598748 PMC8494195

[40] Huang KF, Liu YL, Wang AH (2005) Cloning, expression, characterization, and crystallization of a glutaminyl cyclase from human bone marrow: a single zinc metalloenzyme. Protein Expr Purif 43: 65–72. 16084398 10.1016/j.pep.2005.02.020

[41] Lohkamp B, Andersen B, Piskur J, Dobritzsch D (2006) The crystal structures of dihydropyrimidinases reaffirm the close relationship between cyclic amidohydrolases and explain their substrate specificity. J Biol Chem 281: 13762–13776. 16517602 10.1074/jbc.M513266200

[42] Martinez-Rodriguez S, Martinez-Gomez AI, Clemente-Jimenez JM, Rodriguez-Vico F, Garcia-Ruiz JM, Las Heras-Vazquez FJ, et al (2010) Structure of dihydropyrimidinase from Sinorhizobium meliloti CECT4114: new features in an amidohydrolase family member. J Struct Biol 169: 200–208. 10.1016/j.jsb.2009.10.013 19895890

[43] Patrick GL (2013) An introduction to medicinal chemistry United Kingdom: Oxford University Press.

[44] Keum YS, Jeong YJ (2012) Development of chemical inhibitors of the SARS coronavirus: viral helicase as a potential target. Biochem Pharmacol 84: 1351–1358. 10.1016/j.bcp.2012.08.012 22935448 PMC7092843

[45] Xu H, Ziegelin G, Schroder W, Frank J, Ayora S, Alonso JC, et al (2001) Flavones inhibit the hexameric replicative helicase RepA. Nucleic Acids Res 29: 5058–5066. 11812837 10.1093/nar/29.24.5058 PMC97556

[46] Shadrick WR, Ndjomou J, Kolli R, Mukherjee S, Hanson AM, Frick DN (2013) Discovering new medicines targeting helicases: challenges and recent progress. J Biomol Screen 18: 761–781. 10.1177/1087057113482586 23536547 PMC4427233

[47] Holland DR, Hausrath AC, Juers D, Matthews BW (1995) Structural analysis of zinc substitutions in the active site of thermolysin. Protein Sci 4: 1955–1965. 8535232 10.1002/pro.5560041001 PMC2142975

[48] Hsieh YC, Chen MC, Hsu CC, Chan SI, Yang YS, Chen CJ (2013) Crystal structures of vertebrate dihydropyrimidinase and complexes from *Tetraodon nigroviridis* with lysine carbamylation: metal and structural requirements for post-translational modification and function. J Biol Chem 288: 30645–30658. 10.1074/jbc.M113.496778 24005677 PMC3798535

[49] Gojkovic Z, Rislund L, Andersen B, Sandrini MP, Cook PF, Schnackerz KD, et al (2003) Dihydropyrimidine amidohydrolases and dihydroorotases share the same origin and several enzymatic properties. Nucleic Acids Res 31: 1683–1692. 12626710 10.1093/nar/gkg258 PMC152861

[50] Hermoso JA (2014) Getting CAD in shape: the atomic structure of human dihydroorotase domain. Structure 22: 179–181. 10.1016/j.str.2014.01.005 24507779

[51] Grande-Garcia A, Lallous N, Diaz-Tejada C, Ramon-Maiques S (2014) Structure, functional characterization, and evolution of the dihydroorotase domain of human CAD. Structure 22: 185–198. 10.1016/j.str.2013.10.016 24332717

