# Supplementary figures and images for: Inhibition of a Putative Dihydropyrimidinase from Pseudomonas aeruginosa PAO1 by Flavonoids and Substrates of Cyclic Amidohydrolases
Source: PLoS One. 2015 May 19;10(5):e0127634. doi: 10.1371/journal.pone.0127634 (PMC4437985; doi:10.1371/journal.pone.0127634)

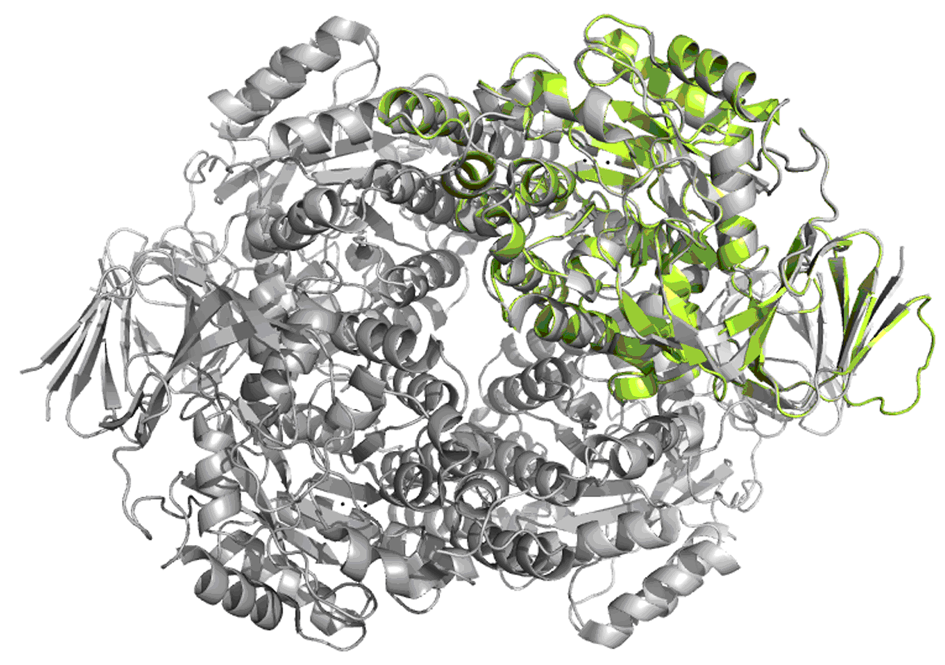

Supplement: S1 Fig — P. aeruginosa dihydropyrimidinase structure was modeled using SWISS-MODEL and human dihydropyrimidinase (PDB entry: 2VR2) as a template. The amino acid residue 3−479 in the modeled structure of P. aeruginosa dihydropyrimidinase (limon) was superimposed with the amino acid residue 5−493 in the crystal structure of human dihydropyrimidinase (gray). A tetrameric structure of human dihydropyrimidinase was shown. (TIF) [file pone.0127634.s001.tif]

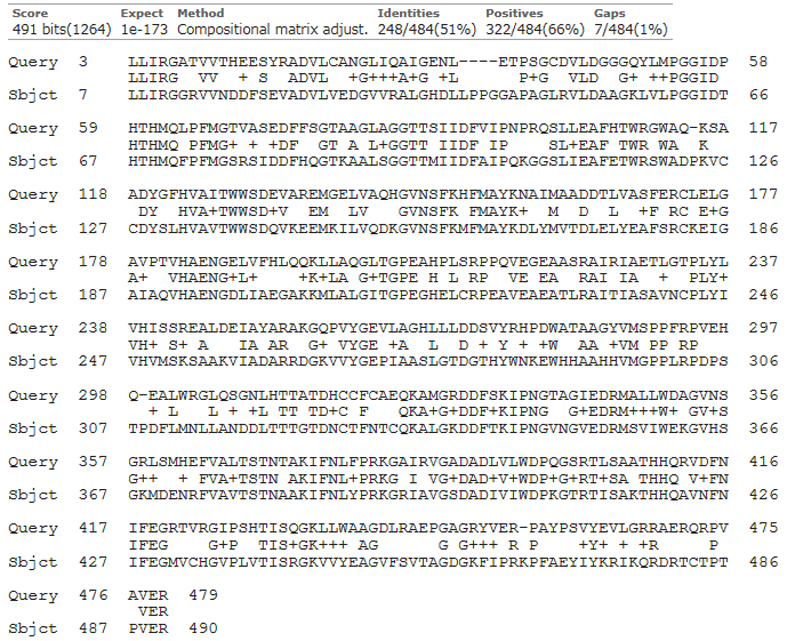

Supplement: S2 Fig — The amino acid sequence of P. aeruginosa dihydropyrimidinase (Query) was aligned with human dihydropyrimidinase (Sbjct). The amino acid sequences of human (with 519 aa) and P. aeruginosa dihydropyrimidinase (with 479 aa) share 51% identity and 66% similarity. (TIF) [file pone.0127634.s002.tif]

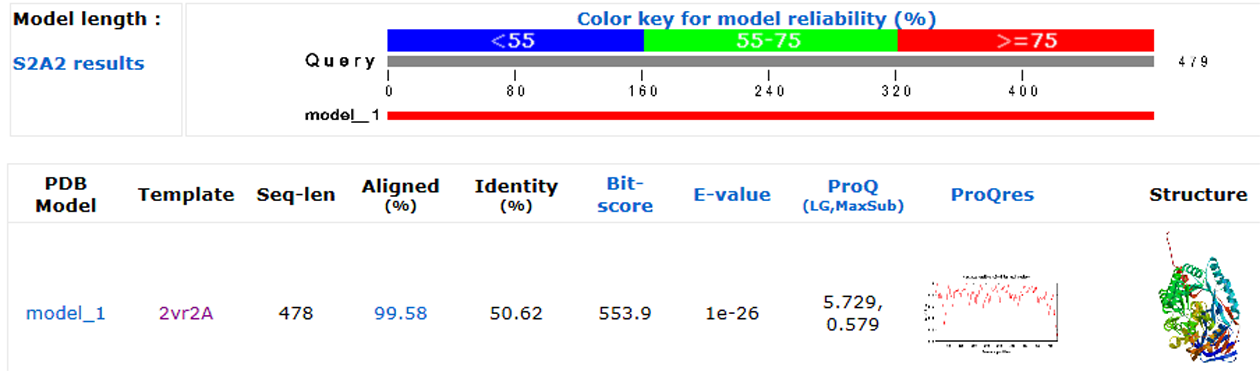

Supplement: S3 Fig — Result from (PS)2 analysis showed that 99.58% of the secondary structure is aligned, indicating a highly similar structure between human and P. aeruginosa dihydropyrimidinase. (TIF) [file pone.0127634.s003.tif]
